# Supplementary material for: Unexpected and Synergistical Effects of All-Trans Retinoic Acid and TGF-β2 on Biological Aspects of 2D and 3D Cultured ARPE19 Cells
Source: Biomedicines. 2024 Sep 30;12(10):2228. doi: 10.3390/biomedicines12102228 (PMC11505250; doi:10.3390/biomedicines12102228)
Supplement: Supplementary file 1 [file biomedicines-12-02228-s001.zip › biomedicines-3213595-supplementary.pdf]

Supplementary File
Table S1

ZO-1	Taqman	GCCACTACAGTATGACCATCC	GCTGGCTTATTCTGAGATGGA	/56-FAM/ACTGAATTA/ZEN/CCTTCACCATGTGCTCCC/3IABkFQ/	NM_175610	4-25
αSMA	Taqman	CTGTTGTAGGTGGTTTCATGGA	AGAGTTACGAGTTGCCTGATG	/56-FAM/AGACCCTGT/ZEN/TCCAGCCATCCTTC/3IABkFQ/	NM_001613	8-9
Col1	Taqman	TTCTGTACGCAGGTGATTGG	GACATGTTCAGCTTTGTGGAC	/56-FAM/TCGAGGGCC/ZEN/AAGACGAAGACATC/3IABkFQ/	NM_000088	1-2a
HIF1α	Taqman	CCGTCATCTGTTAGCACCAT	GCTCACCATCAGTTATTTACGTG	/56-FAM/TCTAGACCA/ZEN/CCGGCATCCAGAAGT/3IABkFQ/	NM_010431	2-3
PGC1α	Taqman	GAGTCTGTTATGGAGTGACATCG	TGTCTGTATCCAAGTCGTTCAC	/56-FAM/ACCAGCCTC/ZEN/TTTGCCCAGATCTTC/3IABkFQ/	NM_013261	1-2
